# Optical coherence tomography findings in acute central retinal artery occlusion and their association with visual outcomes

**DOI:** 10.1186/s40942-025-00770-6

**Published:** 2025-12-11

**Authors:** Hao Wang, Hongyu Wei, Jieying Dong, Fen Zhang, Mei Jiang, Yongye Chang, Ruicong Wang, Rong Yang, Suxia Yan, Zhimin Gao, Liping Zhu, Huijing Sun, Lihui Jin, Minglian Zhang

**Affiliations:** 1 Tianjin Medical University Eye Hospital/Eye Institute, Tianjin Medical University, Tianjin City, 300384 China; 2 Department of TCM Ophthalmology, Hebei Eye Hospital, North China University of Science and Technology, Xingtai City, Hebei Province 054001 China; 3 Hebei Provincial Hospital of Traditional Chinese Medicine Affiliated to Hebei University of Chinese Medicine, Shijiazhuan City, Hebei Province 050017 China; 4 Xiangya Changde Hospital, Changde City, Hunan Province 415009 China; 5 Florence Nightingale Faculty of Nursing, Midwifery & Palliative Care, King’s College London, London, SE1 8WA UK

**Keywords:** Central retinal artery occlusion, Optical coherence tomography, Visual prognosis, Retinal thickness, Optical intensity

## Abstract

**Objectives:**

This study aimed to summarize the optical coherence tomography (OCT) findings in central retinal artery occlusion (CRAO) and investigate their association with visual outcomes.

**Method:**

In this retrospective study, 63 cases (63 eyes) of CRAO were included. Clinical data, including age, gender, time of symptom onset, visual acuity (VA) and OCT images were collected and analysed. The morphology and reflectivity of different retinal layers on OCT images were assessed, and retinal thickness was measured using the built-in software of OCT system. The optical intensity of the retina was quantified using ImageJ software.

**Results:**

The OCT findings in the 63 CRAO eyes were characterized by hyperreflectivity in the inner retinal layers (62 eyes, 98.4%) and retinal thickening (61 eyes, 96.8%), along with prominent middle limiting membrane (p-MLM) (42 eyes, 66.7%), hyporeflectivity of para-foveal outer retina (41 eyes, 65.1%), indistinct inner retinal layers (29 eyes, 46.0%), and foveal deformation (26 eyes, 41.3%). The inner and full retinal thickness in CRAO eyes was moderately positively correlated with the logarithm of the minimum angle of resolution (logMAR) VA at initial presentation and the last follow-up. Additionally, the optical intensity of the outer nuclear layer (ONL) and ellipsoid zone (EZ)-RPE complex was moderately negatively correlated with logMAR VA at presentation and the last follow-up. The ratio of inner retinal optical intensity to EZ/RPE optical intensity was moderately positively correlated with logMAR VA.

**Conclusion:**

OCT may assist in assessing visual outcomes in patients with CRAO. Retinal thickening and inner retinal hyperreflectivity are key OCT findings in CRAO, and these changes could provide valuable prognostic information.

## Introduction

Central retinal artery occlusion (CRAO) is an ophthalmic emergency, which can cause catastrophic visual loss. Animal studies have shown that irreversible retinal damage begins to occur 90 min after CRAO onset, and severe retinal damage develops after 4 h [1–3]. However, in clinical practice, few patients present to hospital within 90 min of CRAO onset, whereas some patients who present beyond 4 h still achieve varying degrees of visual recovery. Currently, various therapies are used to treat CRAO, including carbogen inhalation, acetazolamide infusion, ocular massage, anterior chamber paracentesis, vasodilator administration, and intravenous or intra-arterial thrombolysis. While all these therapies are theoretically beneficial, none has been proven to effectively alter the visual prognosis of patients with CRAO. It appears that regardless of the therapy received, approximately 20%-30% of CRAO patients achieve a final visual acuity (VA) of 20/200 or better [4–8]. Even among patients who received no treatment other than ocular massage, 29% maintained a final VA of ≥ 20/200 [5].

Existing literature indicates that visual acuity (VA) may recover spontaneously or following treatment in some patients with CRAO, whereas even the most timely and aggressive interventions fail to reverse visual impairment in some others. Thus, it is critical to identify patients with different visual prognosis. Optical coherence tomography (OCT) is a rapid, non-contact imaging modality that enables high-resolution in vivo cross-sectional imaging of the retina. It is now widely utilized in clinical ophthalmic practice [9, 10]. In recent years, many studies have examined patients with RAO using OCT [11–16]. In OCT images, CRAO is characterized by thickening and edema of retinal layers in the macular and posterior pole regions [17]. The visual prognosis was shown to be correlated with the macular retinal thickness [16, 18]. Chen et al. [12] also reported the correlation of optical intensity on OCT and visual outcome in CRAO. In the present study, we further summarized the OCT findings in patients with acute CRAO and investigated the association between these findings and visual prognosis.

## Materials and methods

This retrospective study was conducted in accordance with the Declaration of Helsinki, and ethical approval was obtained from the Medical Ethics Committee of Hebei Eye Hospital.

A total of 63 patients with acute CRAO were included, all of whom presented to Hebei Eye Hospital (Xingtai City, Hebei Province, China) between October 2014 and December 2016. The inclusion criteria were as follows: (1) acute unilateral visual decrease lasting less than 1 week; (2) retinal manifestations consistent with CRAO, including ischemic retinal whitening, a cherry-red spot at the macula, attenuated retinal arteries or veins, delayed filling of retinal vessels, and prolonged retinal arteriovenous circulation on fundus fluorescein angiography (FFA); (3) absence of a cilioretinal artery; (4) no other ocular diseases, such as retinal vein occlusion, age related macular degeneration, glaucoma, diabetic retinopathy, et al.; (5) follow-up duration of more than 2 weeks.

All patients included in this study underwent comprehensive ophthalmic evaluations, including assessments of best-corrected visual acuity (BCVA), non-contact tonometry, slit-lamp biomicroscopy, fundus photography, FFA, and OCT. All patients received emergent conservative therapy, including oxygen inhalation, retrobulbar injection of atropine, and intravenous administration of vasodilators. The baseline characteristics of the patients are presented in Table 1. There is no statistical difference in the baseline characteristics between CRAO patients with final BCVA <20/200 and ≥ 20/200 except for the Snellen BCVA at presentation. The follow-up last from 2 weeks to 2 months, during which no complication developed in the patients.

**Fig. 1:**
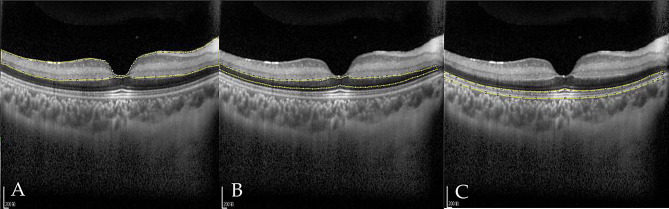
Delineation of regions of interest on OCT. **A**: Inner retina, comprising the nerve fiber layer, ganglion cell layer, inner plexiform layer, inner nuclear layer, and outer plexiform layer; **B**: Outer nuclear layer; **C**: Ellipsoid zone to RPE complex

OCT examinations were performed using either the Spectralis OCT (Heidelberg Engineering Inc., Heidelberg, Germany) or the RTVue100 OCT (Optovue, Fremont, CA, USA). The macula was scanned using a raster scan protocol. CRAO-related OCT changes were determined by comparing the affected eye with the contralateral (unaffected) eye. To assess retinal ischemic edema, full retinal thickness, inner retinal thickness and optical intensity were measured. Subfoveal and parafoveal retinal thickness—defined as thickness at 1000 μm temporal or nasal to the fovea—was measured using the built-in software of the OCT device. Optical intensity refers to the gray level of selected regions, with a scale ranging from 0 (pure black) to 255 (pure white). According to the method described by Chen et al. [12], three regions of interest (ROIs) were defined (Fig. 1): the inner retinal layers (from the retinal nerve fiber layer (RNFL) to the outer plexiform layer (OPL)), the outer nuclear layer (ONL), and the ellipsoid zone (EZ) - retinal pigment epithelium (RPE) complex. The full retina was defined as the layers spanning from RNFL to RPE. The optical intensity of each ROI was measured and quantified using ImageJ software (Version 1.52a, National Institutes of Health, USA) in grayscale JPEG images exported from the OCT software. A single experienced ophthalmologist (H.W.) reviewed and measured all OCT images. The intra-rater reliability was quantified, with all Kappa coefficients (for categorical variables) and intraclass correlation coefficients (for continuous variables) above 0.8.

Statistical analysis were performed using SPSS Version 18.0 (IBM SPSS Statistics, Chicago, IL, USA) or GraphPad Prism 9.0 (GraphPad Software, Inc., San Diego, CA, USA). Continuous data were presented as mean ± standard deviation (SD) or median (range), and compared using t-tests, the Mann-Whitney U test and Wilcoxon signed-rank test. Categorical data were described as frequencies and percentages, and compared using Pearson’s chi-square test or Fisher’s exact test. The correlation between LogMAR VA and retinal thickness or optical intensity was analyzed using Spearman’s rank correlation analysis. The prognostic value of retinal thickness for visual outcome was assessed using receiver operating characteristic (ROC) curve analysis. Final Snellen BCVA was dichotomized at the cutoff point of 20/200 (≥ 20/200 vs. <20/200), as patients with BCVA worse than 20/200 typically experience substantial daily life limitations. The area under the curve (AUC) was used as a global measure of predictive accuracy. All p-values were two-tailed, and a p-value < 0.05 was considered statistically significant.

**Table 1:** Demographic and clinical characteristics of included 63 CRAO patients

		Final snellen BCVA		Total (*n* = 63)	χ^2^	*p* value
		<20/200 (*n* = 39)	≥ 20/200 (*n* = 24)			
Gender	Male	26 (66.7)	14 (58.3)	40 (63.5)		
	Female	13 (33.3)	10 (41.7)	23 (36.5)	0.445	0.505
Age	≤ 45	9 (23.1)	3 (12.5)	12 (19.0)		
	45–60	14 (35.9)	6 (25.0)	20 (31.7)		
	>60	16 (41.0)	15 (62.5)	31 (49.2)	2.821	0.244
Systemic condition	Hypertension	27 (69.2)	15 (62.5)	42 (66.7)	0.303	0.582
	Diabetes	6 (15.4)	6 (25.0)	12 (19.0)	0.891	0.345
	CHD	2 (5.1)	1 (4.2)	3 (4.8)		0.678*
	CVD	13 (33.3)	7 (29.2)	20 (31.7)	0.119	0.730
Time of symptom onset	≤ 24 h	19 (48.7)	10 (41.7)	29 (46.0)		
	24–72 h	11 (28.2)	9 (37.5)	20 (31.7)		
	4–7 days	9 (23.1)	5 (20.8)	14 (22.2)	0.598	0.741
Snellen BCVA at presentation	≤FC	36 (92.3)	10 (41.7)	46 (73.0)		
	0.01–0.1	3 (7.7)	9 (37.5)	12 (19.0)		
	≥ 0.1	0 (0)	5 (20.8)	5 (7.9)		0.001*

CHD: coronary heart disease; CVD: cerebral vascular disease; BCVA: best corrected visual acuity

*: Fisher’s exact test is used

## Results

**Fig. 2:**
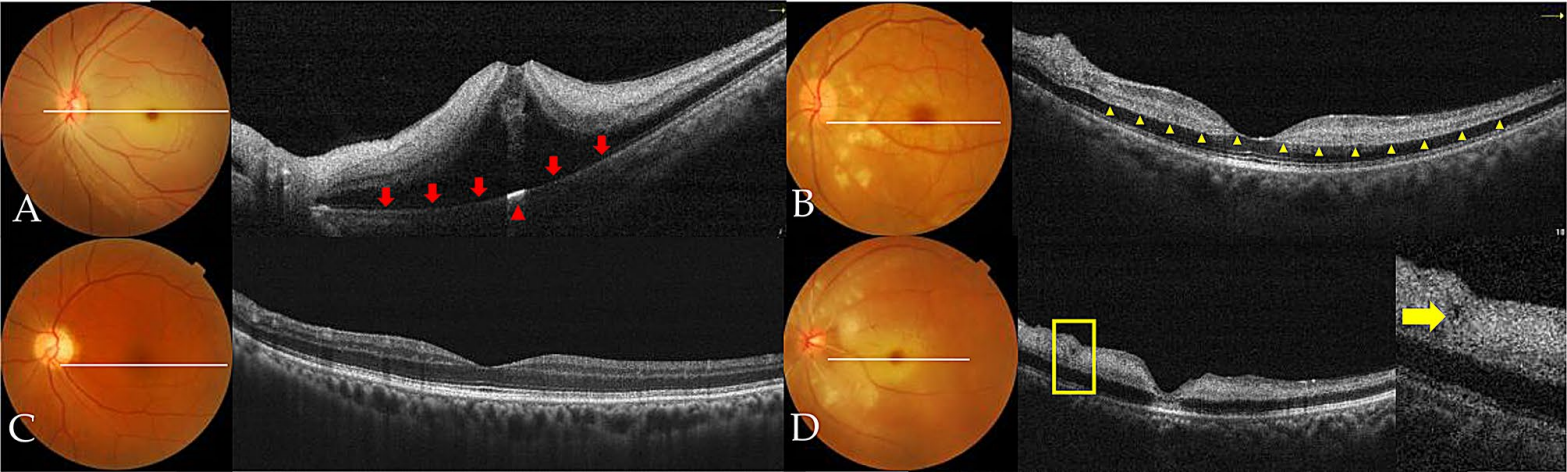
Representative OCT findings in CRAO. **A**: Thickening, hyperreflectivity, and disorganization of the inner retinal layers, accompanied by macular elevation and foveal deformation. These overlying changes result in attenuation of RPE reflectivity (marked by the red arrow) and a subfoveal transmission window (marked by the red arrowhead). **B**: Hyperreflectivity of INL and p-MLM (indicated by the yellow arrowhead). **C**: Retinal thinning without significant hyperreflectivity. The corresponding color fundus photograph shows attenuated retinal vessels. **D**: A small cystic cavity is observed nasal to the fovea

The major OCT findings in the 63 CRAO patients (63 eyes) of this study included retinal thickening, hyperreflectivity of the inner retinal layers, disorganization of the retinal layered structure, foveal deformation, and shadowing of the outer retinal layers (see Fig. 2; Table 2). However, there was 1 case characterized by funduscopic findings of retinal vascular narrowing and fluorescein angiographic evidence of delayed arterial filling, whereas OCT imaging of the affected eye showed retinal thinning without marked hyperreflectivity (see Fig. 2C). In another affected eye, OCT revealed only segmental hyperreflectivity in the inner retinal layers, with no noticeable retinal thickening. There is no statistically significant difference in OCT morphological changes between CRAO patients who present within 24 h of symptom onset and those who present beyond 24 h (see Table 2).

**Fig. 3:**
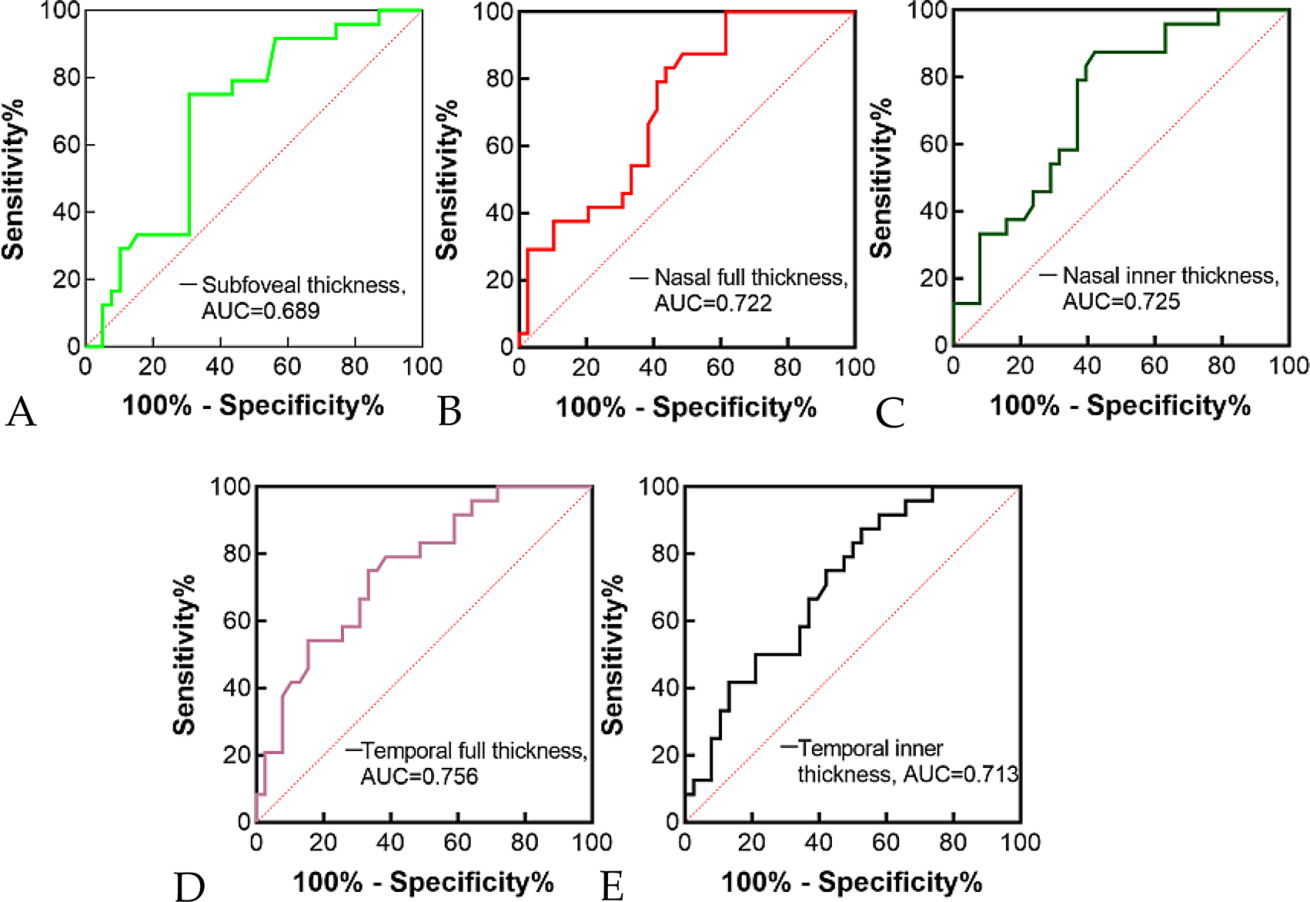
ROC curve of retinal thickness. (**A**) ROC of subfoveal thickness, AUC=0.689; (**B**) ROC of nasal full thickness, AUC=0.722; (**C**) ROC of nasal inner thickness, AUC=0.725; (**D**) ROC of temporal full thickness, AUC=0.756; (**E**) ROC of temporal inner thickness, AUC=0.713

Upon presentation, CRAO eyes exhibited varying degrees of retinal thickening compared to the contralateral healthy eyes, with the most prominent changes observed in the parafoveal region. Both of the full and inner retinal thickness in CRAO eyes was significantly greater than that in the contralateral healthy eyes (Table 3). Parafoveal retinal thickening frequently led to morphological alterations of the fovea, characterized by a shallower or steeper foveal pit, or even complete loss of the foveal depression (Fig. 2B). Pearson’s chi-square test indicated a significant association between the presence of foveal deformation and visual outcome in CRAO eyes (χ² = 13.239, *p* < 0.001). CRAO eyes exhibiting foveal deformation had a poorer visual outcome. In cases with severe ischemic edema, OCT-detected retinal thickening was more pronounced, often accompanied by an undulating retinal surface and obliteration of the foveal contour. Notably, foveal elevation with serous retinal detachment was observed in 4 CRAO-affected eyes (6.3%, Fig. 2A), while cystic cavities within the inner nuclear layer (INL) were identified in 1 eye (Fig. 2D). Spearman’s rank correlation analysis revealed a moderate positive correlation between retinal thickness in the foveal and parafoveal regions and logMAR VA at both initial presentation and final follow-up (Table 5). ROC curve analysis revealed that retinal thickness demonstrated a statistically significant yet modest predictive ability for distinguishing between patients with BCVA ≥ 0.1 and those with BCVA < 0.1. The AUC values ranged from 0.689 to 0.756 (all p-values < 0.05), indicating a fair to moderate level of discrimination (see Fig. 3).

**Table 2:** OCT morphological findings in CRAO

	Total (*n* = 63)	BCVA at last follow-up		χ^2^ value	*p* value	Time of symptom on-set		χ^2^ value	*p* value
		> 20/200 (*n* = 39)	≤ 20/200 (*n* = 24)			≥ 24 h (*n* = 29)	< 24 h (*n* = 34)		
Retinal thickening	61 (96.8)	39 (100)	22 (91.7)		0.141*	29 (100)	32 (94.1)		0.540*
Inner retinal hyperreflectivity	62 (98.4)	39 (100)	23 (95.8)		0.381*	29 (100)	33 (97.1)		0.287*
Foveal deformation	26 (41.3)	23 (59.0)	3 (12.5)	13.239	<0.001	13 (44.8)	13 (38.2)	0.281	0.596
Hyporeflectivity of parafoveal outer retina	41 (65.1)	33 (84.6)	8 (33.3)	17.193	<0.001	20 (72.4)	21 (58.8)	1.272	0.259
p-MLM	42 (66.7)	21 (53.8)	21 (87.5)	7.572	0.006	18 (62.1)	23 (67.6)	0.214	0.643
Obscuration of the inner retinal layered structure	29 (46.0)	26 (66.7)	3 (12.5)	17.547	<0.001	14 (48.3)	15 (44.1)	0.109	0.741

*: Fisher’s exact test is used

Hyperreflectivity of the inner retinal layers was another characteristic OCT finding in CRAO eyes. In normal retinas, the RNFL, IPL, and OPL appear hyperreflective on OCT, with the RNFL exhibiting the highest reflectivity, while the ganglia cell layer (GCL), INL, and ONL are typically hyporeflective. Among the 63 CRAO eyes included in this study, abnormal hyperreflectivity in the inner retinal layers was observed in 62 eyes (98.4%). In cases with severe retinal ischemic edema, OCT revealed diffuse hyperreflectivity throughout the inner retinal layers, which led to obscuration of the retinal layered structure. The shadowing effect caused by the edematous, hyperreflective inner retinal layers resulted in attenuation or even disruption of the signal from the underlying EZ/RPE complex. However, at the site of the cherry-red spot, the fovea act as a transmission window, enabling the hyperreflective RPE band to remain visible (Fig. 2A).

In normal eyes, the OPL is typically visualized on OCT as a moderately reflective band with indistinct boundaries relative to the adjacent retinal layers. However, in 42 of the included CRAO eyes (66.7%), a distinct, well-defined linear hyperreflectivity was observed between the INL and ONL (Fig. 2B), which was named “prominent middle limiting membrane (p-MLM)” by Chu and colleagues [14]. Among the 21 CRAO eyes that did not exhibit the p-MLM, 18 presented with severe retinal edema.

Pearson’s chi-square test showed that the following OCT findings were correlated with visual outcome of CRAO eyes: foveal deformation, hyporeflectivity of parafoveal outer retina, p-MLM, obscuration of the inner retinal layered structure (Table 2).

**Table 3:** Changes of retinal thickness in CRAO

		CRAO eyes	Normal eye	Z value	*p* value
Fovea centralis (um)	Range	187–895	192–249		
	Mean ± SD	280.3 ± 124.5	219.2 ± 14.6		
	Median	236	220	3.912	<0.001*
Nasal side					
Full(um)	Range	311–854	290–400		
	Mean ± SD	435.9 ± 108.1	340.2 ± 21.7		
	Median	409	337	5.397	<0.001*
Inner(um)	Range	127–473	136–203		
	Mean ± SD	249.1 ± 88.4	167.4 ± 18.5		
	Median	215.5	165	3,702	<0.001*
Temporal side					
Full(um)	Range	292–777	282–372		
	Mean ± SD	405.2 ± 94.9	328.1 ± 19.9		
	Median	374	331	5.164	<0.001*
Inner(um)	Range	114–404	126–188		
	Mean ± SD	216.3 ± 67.0	155.7		
	Median	198	158	3.421	0.001*

*: Group comparisons were performed using the Wilcoxon signed-rank test

Compared to normal eyes, CRAO eyes exhibited a significant increase in the optical intensity of the inner retinal layers (t = -3.067, *p* = 0.009). Conversely, the optical intensity of the ONL and the EZ/RPE complex were significantly reduced (Table 4). The ratio of inner retinal optical intensity to EZ/RPE complex optical intensity was also significantly increased (t = -6.351, *p* < 0.001). Spearman’s rank correlation analysis revealed no statistically significant correlation between the optical intensity of the inner retinal layers in CRAO eyes and logMAR visual acuity at either initial presentation or final follow-up. In contrast, the optical intensity values of both the ONL and the EZ/RPE complex showed a negative correlation with logMAR visual acuity at both initial and final follow-up visits. Furthermore, the inner retina-to-EZ/RPE optical intensity ratio demonstrated a positive correlation with logMAR VA at both time points (Table 5).

**Table 4:** Optical intensity of the retina in eyes with CRAO

	Optical intensity		
	Inner retina	ONL	EZ/RPE
Normal eye	126.0 ± 15.0	66.1 ± 12.0	177.2 ± 12.1
CRAO eye	148.4 ± 18.9	50.7 ± 16.2	104.6 ± 34.5
t value	-3.067	2.477	8.382
*p* value	0.009	0.028	<0.001

*Group comparisons were performed using the Student’s t test

**Table 5:** Correlations between retinal thickness, optical intensity, and LogMAR VA in CRAO

		LogMAR VA at presentation		Final LogMAR VA	
		Correlation coefficient	*p* value	Correlation coefficient	*p* value
Full retinal thickness	Fovea	0.552	<0.001	0.408	<0.001
	Nasal	0.582	<0.001	0.453	<0.001
	Temple	0.630	<0.001	0.547	<0.001
Inner retinal thickness	Nasal	0.531	<0.001	0.436	<0.001
	Temple	0.582	<0.001	0.495	<0.001
Optical intensity	Inner retina	-0.064	0.618	0.055	0.670
	ONL	-0.419	0.001	-0.323	0.01
	EZ-RPE	-0.617	<0.001	-0.550	<0.001
	Inner retina / EZ-RPE ratio	0.586	<0.001	0.552	<0.001

## Discussion

This study retrospectively analyzed OCT images from 63 patients with CRAO who presented within 1 week of symptom onset. The major OCT findings included retinal thickening and inner retinal hyperreflectivity, along with foveal deformation, hyporeflectivity of parafoveal outer retina, p-MLM, obscuration of the inner retinal layered structure. Quantitative assessment of retinal thickness revealed a moderate positive correlation between inner and full retinal thickness in the foveal and parafoveal regions and logMAR visual acuity at both initial presentation and final follow-up. Furthermore, the optical intensity values of the ONL and the EZ/RPE complex showed a moderate negative correlation with logMAR visual acuity, whereas the ratio of inner retinal optical intensity to EZ/RPE optical intensity demonstrated a moderate positive correlation with logMAR visual acuity at both time points.

The normal retina is characterized by a transparent, layered structure. During OCT examination, each retinal layer reflects, transmits, and absorbs the scanning beam differently, producing the typical layered pattern with varying reflectivity on OCT. In CRAO, cells in the inner retina develop intracellular edema, which leads to tissue swelling and reduced translucency. This manifests as retinal opacity on funduscopy and corresponds to inner retinal thickening and hyperreflectivity on OCT. The foveola remains transparent due to its choroidal blood supply, which accounts for the classic cherry-red spot observed on funduscopy and the subfoveal EZ/RPE hyperreflectivity on OCT. The majority of CRAO eyes exhibit these changes to varying degrees at initial presentation. Retinal edema represents the core pathological process in CRAO. Several studies have reported an association between retinal edema and visual outcomes in CRAO [11, 19]. In this context, OCT is currently the optimal modality for the objective assessment of retinal edema in CRAO.

This study evaluated the role of OCT in CRAO from both qualitative and quantitative perspectives to assess retinal edema. We found that foveal morphological abnormalities, signal attenuation of the outer retinal layers, and disorganization of the retinal layered structure—all caused by ischemic retinal edema—were associated with visual prognosis. CRAO eyes with these changes had a poorer visual outcome. Furthermore, the relationship between retinal edema and visual prognosis in CRAO was further validated via quantitative measurements of inner and full retinal thickness, and optical intensity. Reports on the relationship between retinal thickness and visual prognosis in CRAO-affected eyes have been inconsistent. Consistent with the findings of Wang et al. [18] and Ahn et al. [11], our results demonstrated a positive correlation between retinal thickness and final logMAR visual acuity. In contrast, Chen et al. [13] reported no such correlation. The discrepancy may be attributable to their considerably smaller sample size (*n* = 11), which limits the statistical power to detect a correlation. In this study, ROC curve analysis for both the inner and full retinal thickness to assess their ability to predict visual prognosis was performmed, and the AUC values ranged from 0.689 to 0.756, which represent a fair to moderate level of discrimination. While the predictive power is moderate, it provides a quantifiable and objective basis for prediction of CRAO visual prognosis. Hopefully, the futural studies could enhance predictive accuracy by combining retinal thickness with other clinical or imaging biomarkers.

Optical intensity on OCT is another valuable metric for assessing ischemic retinal edema. CRAO typically increases the optical intensity of the inner retinal layers while concurrently decreasing the optical intensity of the ONL and EZ/RPE complex [20]. The absolute optical intensity value is susceptible to variations in incident scanning beam intensity and can be affected by factors such as ocular media opacities, OCT device light source power, eye position during image acquisition, and pupil size. To mitigate these confounding variables, the concept of “optical intensity ratio” (OIR) has been proposed [21]. The OIR captures both the reflective and transmissive properties of the inner retina simultaneously, suggesting its potential as a useful index for quantifying pathological changes in this region. Both the present study and the report by Chen et al. [12] consistently demonstrated a positive correlation between the inner retina-to-EZ/RPE complex OIR and final logMAR visual acuity in CRAO eyes.

Furthermore, this study identified an association between the p-MLM in CRAO eyes and visual prognosis. In the normal retina, the OPL presents as a band of moderate reflectivity with indistinct borders. In CRAO, a distinct linear hyperreflectivity can be observed within the OPL. This feature wastermed the “prominent middle limiting membrane (p-MLM)” [14]. Its pathological basis is likely due to swollen synaptic terminals of bipolar cells within the OPL, rather than a true membranous structure. The presence of p-MLM serves as a sensitive indicator of retinal ischemia. In our clinical practice, we have observed CRAO cases where the INL itself showed no significant hyperreflectivity, yet a p-MLM was already evident in the subjacent OPL. Previous studies have reported that CRAO-affected eyes exhibiting this interlayer hyperreflectivity are associated with a more favorable visual prognosis [22]. Our findings are consistent with this observation. We hypothesize that in CRAO eyes without p-MLM, retinal edema is so severe that the consequent shadowing or obscuration effect from the edematous, hyperreflective inner retina may mask the p-MLM, precluding its visualization.

The present study has several limitations. First, its retrospective, single-center design with a limited sample size may have affected the generalizability of our findings and precluded more complex multivariate analyses. Second, the relatively short follow-up period prevented the assessment of long-term visual and structural outcomes beyond the acute phase. Therefore, our conclusions warrant validation in larger, prospective, and multi-center cohorts with extended follow-up.

## Conclusions

In summary, this study demonstrates that OCT can serves as a crucial tool for diagnosing CRAO, and that specific alterations on OCT show a significant association with visual outcome, providing valuable prognostic information.

## Data Availability

The data used to support the findings of this study are available from the corresponding author on request.

## Abbreviations

AUC: Area under the curve
BCVA: Best corrected visual acuity
CHD: Coronary heart disease
CRAO: Central retinal artery occlusion
CVD: Cerebral vascular disease
EZ: Ellipsoid zone
GCL: Ganglia cell layer
INL: Inner nuclear layer
logMAR: Logarithm of the minimum angle of resolution
OCT: Optical coherence tomography
ONL: Outer nuclear layer
ONL: Outer nuclear layer
OPL: Outer plexiform layer
p-MLM: Prominent middle limiting membrane
RNFL: Retinal nerve fiber layer
ROC curve: Receiver operating characteristic curve
ROI: Regions of interest
RPE: Retinal pigment epithelium
VA: Visual acuity
